# Supplementary material for: Protective Effects of Velvet Antler Methanol Extracts on Hypoxia-Induced Damage in Caenorhabditis elegans through HIF-1 and ECH-8 Mediated Lipid Accumulation
Source: Nutrients. 2024 Jul 13;16(14):2257. doi: 10.3390/nu16142257 (PMC11280314; doi:10.3390/nu16142257)
Supplement: Supplementary file 1 [file nutrients-16-02257-s001.zip › nutrients-3082364-supplementary.pdf]

Supplementary Materials

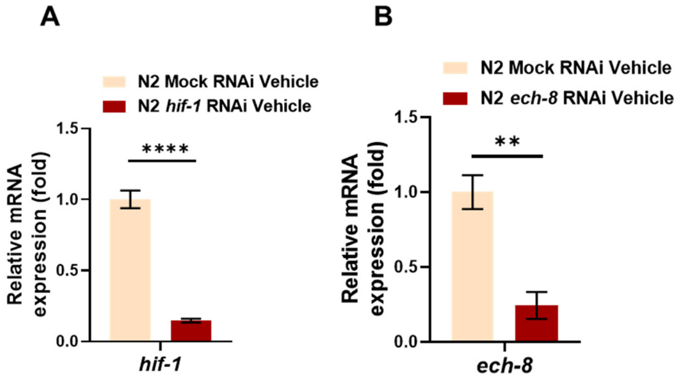

Figure S1. The RNAi efficiency measured by qPCR. The expression of *hif-1* (A) and *ech-8* (B) genes in RNAi experiments. The worms' RNAi feeding was initiated at the L1 stage.

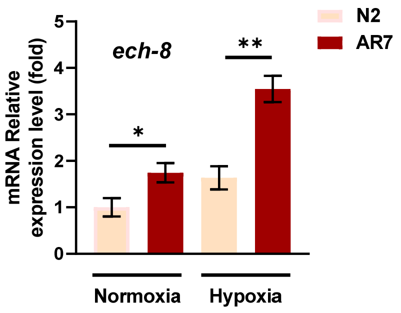

Figure S2. The mRNA of *ech-8* expression measured by qPCR. N2 and AR7 (*hif-1* mutant) worms were treated with or without 0.4 mg/mL MEs under normoxia or hypoxia conditions. Error bars represented the SEM of three independent replicates. *, $p < 0.05$; **, $p<0.01$.
